# Silica-ZnCl_**2**_: An Efficient Catalyst for the Synthesis of 4-Methylcoumarins

**DOI:** 10.1155/2013/132794

**Published:** 2013-12-16

**Authors:** Bandita Datta, Mohamed Afzal Pasha

**Affiliations:** Department of Studies in Chemistry, Central College Campus, Bangalore University, Bengaluru 560001, India

## Abstract

Silica-ZnCl_2_ has been found to be an efficient and eco-friendly catalyst for the synthesis of substituted 4-methylcoumarins from ethyl acetoacetate and substituted phenols under “neat” conditions in an oil bath at 80°C. The experimental procedure is simple, includes shorter reaction times (15–65 min), compatible with sensitive functional groups, and results in excellent yield of the products.

## 1. Introduction

Since long time, the use of solid phase technique has been considered to be the method of choice for application in a large number of diverse and interesting chemical manipulations. Reactions involving solid-supported catalysts encompass advantages like easy work up of the reaction by filtration of solid-supported reagent, increased reactivity, higher yields of the product, and recyclability of the reagent for subsequent reaction [1]. The utility of the solid-supported reactions has been acknowledged in the synthesis of several chemical libraries. A large number of solid-supported reagents like SiO_2_-SO_3_H [2], PPA-SiO_2_ [3], and HClO_4_-SiO_2_ [4] have been developed over the period of time and have been used in many chemical reactions. In our laboratory we have developed many silica-based reagents [5], which fascinated us for the development of other solid-supported reagents.

ZnCl_2_, being an inexpensive and easily available catalyst, has been demonstrated in chemical reactions as a Lewis acid catalyst [6]. Although it is a user choice catalyst, it is very hygroscopic accompanied with its nonusability. Therefore, we have prepared a silica-ZnCl_2_ reagent by Paul's method [7] which can find application as a user-friendly catalyst. To check its applicability, we wanted to use this catalyst in the preparation of biologically important molecules, whose synthetic preparation can be improved. Hence, we chose the synthesis of substituted coumarins which have a long list of applications.

Coumarins are found to have varied bioactivities including inhibition of platelet aggregation [8], inhibition of steroid 5a-reductase [9], inhibition of HIV-1 protease [10], and antibacterial [11] and anticancer [12] activities. Suitably substituted coumarins have various applications in food additives, pharmaceutical, perfume, and cosmetic industries [13]. Moreover, 7-hydroxy-4-methylcoumarin derivatives have also been used in the synthesis of dendrimers [14]. Since coumarins have become one of the prime molecules of investigation, several groups have started synthesizing molecules with similar moieties for further applications.

Coumarins can be synthesized using several strategies like the Pechmann reaction, Perkin reaction, and Knoevenagel condensation. The widely used scheme is of Pechmann using ethyl acetoacetate and a phenol in the presence of concentrated sulfuric acid as a catalyst [15]. Several other catalysts have also been used including ZnCl_2_ [16] which involves harsh conditions, requires longer time, gives lesser yields, and involves tedious workup procedure. Hence, a user-friendly protocol for the synthesis of coumarins needs to be developed. In our laboratory we have been synthesizing synthons, which are used in the synthesis of biologically important molecules like **β**-enaminones [17], nitriles [18], formamides [19], and so on. In this report, we are presenting a simple and efficient procedure for the synthesis of substituted 4-methylcoumarins using silica-supported ZnCl_2_ as a heterogeneous catalyst which can acknowledge the advantages of solid-supported catalysts.

## 2. Experimental

### 2.1. Materials and Methods

Ethyl acetoacetate and substituted phenols were commercial products and were used without further purification. Yields refer to yield of the isolated products. Melting points were measured on a RAAGA Indian made melting point apparatus; GC-mass spectra were recorded on a Shimadzu GC-MS QP 5050A instrument.

### 2.2. Preparation of SiO_2_-ZnCl_2_

To a mixture of anhydrous ZnCl_2_ (3 g) and activated SiO_2_ (10 g) in a 100 mL round bottomed flask, DCM (30 mL) was added and the reaction mixture was refluxed for 10 h. SiO_2_-ZnCl_2_ was obtained as a free-flowing powder after filtration under reduced pressure and dried at 110°C for 12 h, and it was stored in a desiccator over P_2_O_5_.

### 2.3. Typical Experimental Procedure

A mixture of ethyl acetoacetate (2 mmol), phenols (2 mmol), and silica-ZnCl_2_ (100 mg) was taken in a 50 mL flat-bottomed flask fitted with a condenser, mixed well, and heated in a preheated oil bath at 80°C for 15–65 min. After completion of the reaction (monitored by TLC), the contents were cooled to 25°C and EtOAc (5 mL) was added to the reaction mixture. The solid catalyst was removed by filtration and washed with dry and warm ethanol and kept aside for reuse. The reaction mixture was washed with water (5 mL) and dried over MgSO_4_. The solvent was removed under vacuum to get the product. The crude product was further purified either by column chromatography or by recrystallization from ethanol : water (1 : 1). Yields and physical constants of all the products prepared by this procedure are presented in Table 2.

## 3. Results and Discussion

**Figure 1:**
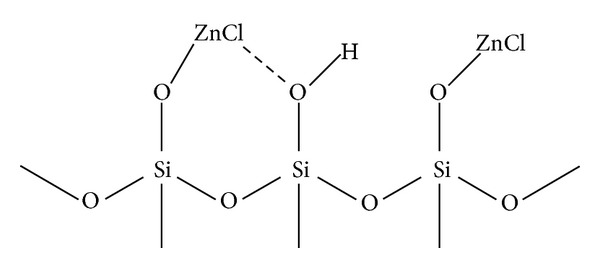
Olson's proposed structure of silica-ZnCl_2_.

As part of our ongoing research program for exploring efficient solid-supported catalysts, we used silica-supported ZnCl_2_ for the synthesis of coumarins. The stability of this catalyst was determined by thermogravimetric analysis (TGA) and found that there is no loss of physiosorbed and chemisorbed ZnCl_2_ from the surface of silica gel and hence the reaction can be carried out at 80°C [20]. On the basis of the elemental analysis of solid-supported ZnCl_2_, the probable structure of the catalyst was reported by Olson et. al. (Figure 1) [21].

**Scheme 1:**
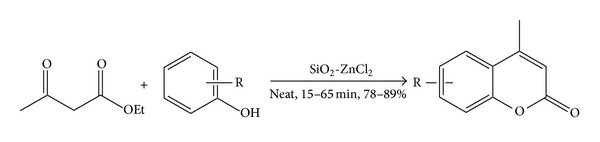
Synthesis of 4-methylcoumarins under neat condition.

We initiated our investigation with the condensation of ethyl acetoacetate and resorcinol in the presence of silica-supported ZnCl_2_ under reflux condition in ethanol. Even after refluxing for 5 h. the desired product was not detected. Hence, we increased the reaction temperature to 80°C and continued the reaction. Though the product was detected, unsatisfactory yield was obtained. In order to obtain the optimum conditions we considered the use of other solvents but obtained poor yields in these solvents. Better result was obtained when the reaction was carried out under solvent-free neat condition (Table 1). We were not very happy with this result also; we still wanted to increase the yield of the product and hence, taking ethyl acetoacetate and resorcinol as a model reaction, we varied the amount of the catalyst used in the reaction. The optimum result was obtained when 100 mg of the catalyst was used. If we increase the quantity to 200 mg no change in the yield of the product is observed, and on reduction to 50 mg poor yield of the desired product was noticed (Scheme 1).

To explore the scope of the optimized reaction conditions we extended our procedure to various substituted phenols in the presence of ethyl acetoacetate. We found that the reaction proceeds very efficiently with all electron-withdrawing groups as well as electron-donating groups present in the nucleus of phenol. We used *α*-naphthol and *β*-naphthol also (Table 2) and both gave good yield of the respective products. All the prepared products were characterized by GC-mass spectral analysis, by comparison of the melting points and comparison on TLC with the standard compounds prepared by reported methods.

**Figure 2:**
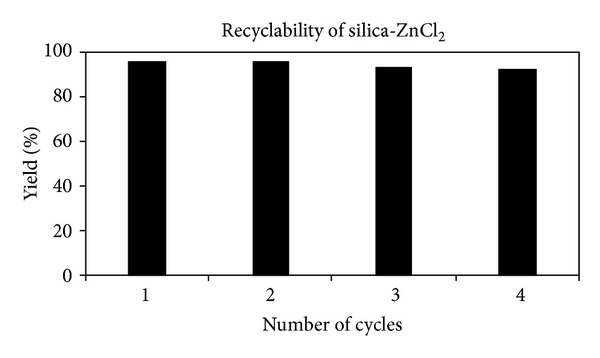
Recyclability of silica-ZnCl_2_ (100 mg) in the present reaction.

An advantage of the solid-supported catalyst is its recyclability. In view of development of eco-friendly methodologies, recovery and reuse of the catalyst is highly preferable. Silica-supported ZnCl_2_ was easily separated from the reaction medium after adding EtOAc (5 mL), washed with dry and warm ethanol, and reused in the subsequent reactions. As indicated in Figure 2 recycled silica-supported ZnCl_2_ showed no loss of efficiency with regard to reaction time, and little loss of yield after four successive runs was noticed which may be due to loss of the catalyst during recovery. The yields of the products for the four cycles are 95, 94, 92, and 92%, respectively.

## 4. Conclusion

In conclusion, we have found that the reaction of ethyl acetoacetate and substituted phenols leads to an efficient synthesis of substituted 4-methylcoumarins in good to very high yields within relatively short reaction times using silica-supported ZnCl_2_ as a simple and reusable heterogeneous catalyst. The reaction is simple, mild, and convenient and is environment friendly.

## Figures and Tables

**Table 1:** Optimization of the reaction conditions using silica-ZnCl_2_.

Entry	Amount of silica-ZnCl_2_ (mg)	Solvents	Temperature (°C)	Time	Yield (%)^a^
1	100	Ethanol	60	5 h	ND
2	100	Ethanol	80	5 h	25
3	100	Methanol	reflux	5 h	Trace
4	100	MeCN	80	5 h	10
5	100	DCM	reflux	5 h	ND
6	100	Neat	80	15 min	95
7	200	Neat	80	15 min	89
8	50	Neat	80	15 min	65

^a^Isolated yields. Reaction conditions: ethyl acetoacetate (2 mmol), resorcinol (2 mmol), solvent (5 mL), and silica-ZnCl_2_.

**Table 2:** Synthesis of coumarin derivatives using ethyl acetoacetate and substituted phenols in the presence of silica-ZnCl_2_.

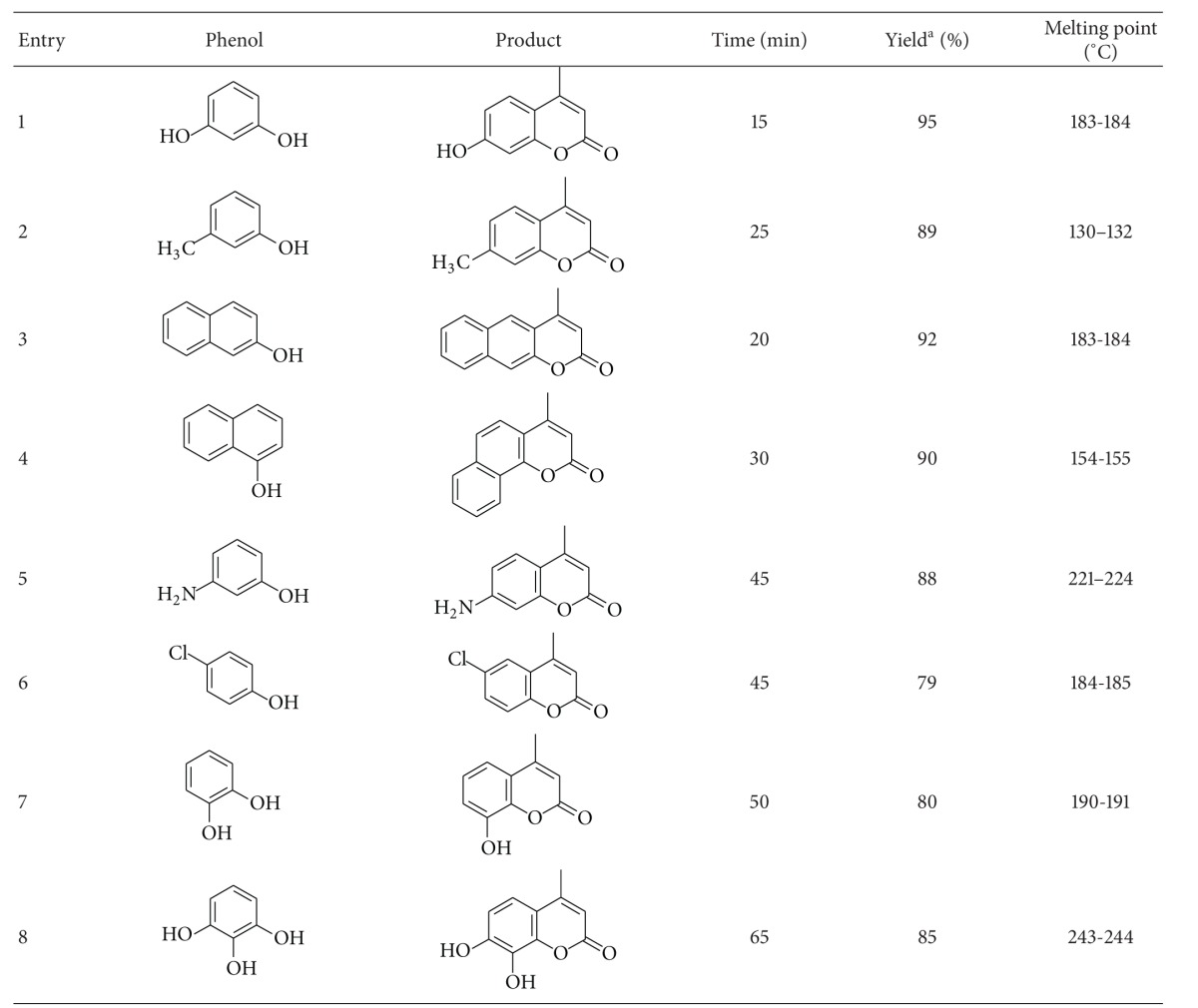

^a^Isolated yields.
